# Self management, joint protection and exercises in hand osteoarthritis: a randomised controlled trial with cost effectiveness analyses

**DOI:** 10.1186/1471-2474-12-156

**Published:** 2011-07-11

**Authors:** Krysia S Dziedzic, Susan Hill, Elaine Nicholls, Alison Hammond, Helen Myers, Tracy Whitehurst, Jo Bailey, Charlotte Clements, David GT Whitehurst, Sue Jowett, June Handy, Rhian W Hughes, Elaine Thomas, Elaine M Hay

**Affiliations:** 1 Arthritis Research UK Primary Care Centre, Keele University, Keele, Staffordshire, ST5 5BG, UK; 2 Centre for Health, Sport & Rehabilitation Research, University of Salford, Frederick Road, Salford, Greater Manchester, M6 6PU, UK; 3 School of Population and Public Health, University of British Columbia, Vancouver, BCV5ZIM9, Canada; 4 School of Health and Population Sciences, University of Birmingham, Edgbaston, Birmingham, B15 2TT, UK

## Abstract

**Background:**

There is limited evidence for the clinical and cost effectiveness of occupational therapy (OT) approaches in the management of hand osteoarthritis (OA). Joint protection and hand exercises have been proposed by European guidelines, however the clinical and cost effectiveness of each intervention is unknown.

This multicentre two-by-two factorial randomised controlled trial aims to address the following questions:

• Is joint protection delivered by an OT more effective in reducing hand pain and disability than no joint protection in people with hand OA in primary care?

• Are hand exercises delivered by an OT more effective in reducing hand pain and disability than no hand exercises in people with hand OA in primary care?

• Which of the four management approaches explored within the study (leaflet and advice, joint protection, hand exercise, or joint protection and hand exercise combined) provides the most cost-effective use of health care resources

**Methods/Design:**

Participants aged 50 years and over registered at three general practices in North Staffordshire and Cheshire will be mailed a health survey questionnaire (estimated mailing sample n = 9,500). Those fulfilling the eligibility criteria on the health survey questionnaire will be invited to attend a clinical assessment to assess for the presence of hand or thumb base OA using the ACR criteria. Eligible participants will be randomised to one of four groups: leaflet and advice; joint protection (looking after your joints); hand exercises; or joint protection and hand exercises combined (estimated n = 252). The primary outcome measure will be the OARSI/OMERACT responder criteria combining hand pain and disability (measured using the AUSCAN) and global improvement, 6 months post-randomisation. Secondary outcomes will also be collected for example pain, functional limitation and quality of life. Outcomes will be collected at baseline and 3, 6 and 12 months post-randomisation. The main analysis will be on an intention to treat basis and will assess the clinical and cost effectiveness of joint protection and hand exercises for managing hand OA.

**Discussion:**

The findings will improve the cost-effective evidence based management of hand OA.

**Trial registration:**

**identifier: **ISRCTN33870549

## Background

Osteoarthritis (OA) is the commonest form of arthritis in the UK. It is the source of most of the musculoskeletal pain and disability in adults aged 50 years and over [1] and the hand is one of the most common sites of pain and osteoarthritic change in this age-group [2,3]. In a large cross-sectional survey of older adults with musculoskeletal hand problems in North Staffordshire, participants reported significant hand pain and disability, which affected their everyday lives [4]. The majority of people with hand OA are managed in primary care but often core treatments recommended by European and UK guidelines are not given [5] and patients report dissatisfaction with management [6]; 'I went to the GP (he) gave me a form...with osteoarthritis or something, whatever they call it. I thought that wasn't very helpful. 'Nothing we can do about it' he said and at the time I'd got really bad pain, which was why I went.... down the thumb. I honestly wouldn't ever go back and tell them my hands are playing up 'cause he said there was nothing they could do' [6]. As a consequence few people with hand problems visit their general practitioner (GP), even when severely affected [4], and even fewer attend for occupational therapy (OT) [7]. In our survey, only 3% of those with severe disability reported seeing an occupational therapist (OT) in the last year [4] despite the fact that OTs commonly deliver core treatments for people with hand OA.

Joint protection and hand exercises are core components of OT. Joint protection aims to reduce pain, disability and improve function through the use of ergonomic approaches such as altering movement patterns, modification of task and environment, and use of assistive devices [8]. Patients are helped to understand how strain on the joint when carrying out daily activities can contribute to joint pain and potentially promote joint deformity. Hand exercises also aim to reduce pain and disability, and improve physical functioning and grip strength [9]. Studies in patients with lower limb OA suggest that exercise therapy may delay or even prevent the onset of disease [10] although its effectiveness in hand OA is still uncertain.

Increasingly, OTs use educational-behavioural approaches to enhance the use of self-management and behaviour change interventions such as exercise and joint protection [8,11,12]. Goal-setting and problem-solving, with adequate time to practice new skills in order to develop new habits and routines, are used to facilitate uptake of exercise and joint protection techniques [11,12].

Despite the fact that joint protection and hand exercises are frequently used by OTs and physiotherapists (PTs) in the management of hand OA, and have been recommended for all patients in the European League Against Rheumatism (EULAR) recommendations [13], systematic reviews conclude there is a paucity of evidence to support these interventions [14-16]. One trial in secondary care demonstrated modest benefits of joint protection plus hand exercises compared with an education leaflet for hand OA [17]. A study of yoga exercises in a hand OA population has shown promising findings [18].

The EULAR recommendation to provide joint protection and hand exercises for all patients with hand OA is based largely on expert opinion and has not been evaluated in high quality randomised controlled trials. The majority of patients with hand OA will be managed in primary care and it is therefore important to evaluate the benefits of hand exercises and joint protection before the EULAR recommendations can be adopted in this setting. This paper outlines the protocol for the Self Management in Osteoarthritis of the Hand (SMOotH) trial.

### Trial development

The trial was designed with key stakeholders: OTs with experience of treating patients with hand OA, and research users with experience of living with or caring for someone with hand OA.

### Occupational Therapists

We have established a clinical advisory group of 10 OTs working in hand therapy and musculoskeletal conditions in North Staffordshire and Central Cheshire, UK. The group helped develop the research questions, interventions and the trial design. We have used this approach successfully in previous studies of physiotherapy [19,20]. The OT clinical advisory group was consulted at all stages of the study development through four half-day workshops, and identified the research questions as important to current clinical practice. These workshops considered the current best evidence for the management of hand OA using critically appraised topics [21].

### User involvement

*In the UK there is a clear policy directive to involve patients and the public in research *[22]. Such involvement is thought to lead to research which is of clinical relevance and of better quality [23-26]. We have an established Research User Group and Virtual User Panel who provide advice and feedback on trial conduct and offer patient representation on the trial steering groups.

We will engage both OTs and Research Users throughout each stage of the trial.

### Trial Objectives

Specifically, our study will consider the following main research questions:

• Is joint protection delivered by an OT more effective in reducing hand pain and disability than no joint protection in people with hand OA in primary care?

• Are hand exercises delivered by an OT more effective in reducing hand pain and disability than no hand exercises in people with hand OA in primary care?

• Which of the four management approaches explored within the study (leaflet and advice, joint protection, hand exercise, or joint protection and hand exercise combined) provides the most cost-effective use of health care resources

These research questions are in line with recommendations of the EULAR guidelines for the management of hand OA [13]. The study has been designed to meet the Osteoarthritis Research Society International (OARSI) recommendations for clinical trials in hand OA [27].

## Methods/Design

This is a multicentre two-by-two factorial randomised controlled trial in community-dwelling older adults of non-pharmacological interventions [28] with a superiority design [29]. Participants will be allocated to one of four groups: leaflet and advice; a joint protection programme; a hand exercise programme; or a joint protection and hand exercise programme (see Table 1).

**Table 1:** Two by two factorial randomised trial: leaflet and advice, joint protection, hand exercises, joint protection and hand exercises

	Leaflet and advice alone	Joint protection
**Leaflet and advice alone**	Leaflet and advice	Leaflet and advice Joint protection
**Hand exercises**	Leaflet and advice Hand exercises	Leaflet and advice Hand exercises and joint protection

Rows and column headings indicate possible interventions, cells indicate the four possible treatment allocations.

### Participants

All participants aged 50 years and over registered with 3 general practices in Central Cheshire and North Staffordshire (estimated n = 9,500) will be mailed a health survey questionnaire asking about their general health and any hand pain or hand problems experienced for a day or longer over the past 12 months. Prior to mailing, general practitioners (GPs) will have the opportunity to screen the participant list for any exclusions e.g. vulnerable adults, those with psychiatric illness. Immediately prior to mailing, a deaths and departure check will be completed to verify that participants are still registered at the GP practice and have not recently died or left the practice. To avoid contamination between participants only one person for each address will be considered eligible for the study. This will avoid any contamination of interventions between individuals in the same household. The first person from the household to respond to the survey will be deemed eligible.

All participants responding to the health survey questionnaire will be screened for eligibility. Those who meet the eligibility screen (see Table 2) will be contacted by post with a letter outlining the trial, a further study information sheet, and an invitation to telephone the research centre should they wish to attend for clinical assessment. Those who wish to take part will be asked to make an appointment to have a brief clinical assessment by a research nurse, undertake a further phase of eligibility screening (see Table 3) and a face-to-face consent procedure. At the end of the clinic, details of eligible participants will be forwarded to the research centre and participants will be randomised to one of four groups: leaflet and advice; joint protection (looking after your joints); hand exercises; or a combined intervention of joint protection and hand exercises.

**Table 2:** Eligibility criteria assessed on the health survey questionnaire

Participants will be eligible to be invited to the baseline nurse clinical assessment if they meet the following criteria on the health survey questionnaire:

• Give consent to further contact
• Report hand pain in the last 12 months
• Report hand pain aching or stiffness on "some days", "most days" or "all days" in the last month
• Have an AUSCAN pain score > = 5 or an AUSCAN function score > = 9
• Report they have not:
a. seen an occupational or physiotherapist for their hand in the last 6 months
b. injured their hands badly enough to see a doctor in the last 6 months
c. had any hand operations in the last 6 months
d. had any hand injections in the last 6 months
• No other household member is participating in the trial

**Table 3:** Eligibility criteria assessed by the research nurse at the baseline nurse clinical assessment

Participants will be randomised to the trial if they meet the following criteria at the baseline nurse clinical assessment:

• Give informed consent to participate in the trial
• Meet the ACR criteria for features of hand OA (symptoms previously assessed on health survey) or have unilateral or bilateral thumb base OA
• Do not have a clinical "red flag" indicative of potentially serious pathology
• Able to attend OT classes

### Eligibility criteria

Participants included in the trial will be aged 50 years and over identified from general practice registers. Eligibility criteria for each stage of the study are based on the recommendations of the OARSI task force on design and conduct of clinical trials in hand OA [27]. Inclusion criteria are: males and females; aged 50 years and over; fulfilling the American College of Rheumatology (ACR) definition of symptomatic hand osteoarthritis [27,30], or symptomatic thumb base OA on clinical assessment; no other household member participating in the trial; ability to understand and capable of giving written informed consent. Exclusion criteria are: consultation or treatment for this hand problem in the previous 6 months including an intra-articular joint injection to wrist, fingers or thumb, fractures or significant injury or surgery to the wrist or hand [27]; consultation for this hand problem with an occupational therapist or physiotherapist; red flags, e.g. history of serious illness or disease (e.g. stroke), progressive neurological signs, acute swollen joint; those with a diagnosis of inflammatory arthritis (e.g. rheumatoid arthritis (RA), psoriatic arthritis); minimal pain and function on the Australian/Canadian hand outcome score (AUSCAN) [31] pain < 5 and function < 9) [27]. Individuals with co-existing hand conditions, such as carpal tunnel syndrome, Dupuytrens contracture, trigger finger, will not be excluded unless the condition is deemed at the clinic to be the primary cause of the hand problem.

### Clinic assessment procedures

#### Invitation to the clinic

Respondents to the health survey questionnaire who meet the eligibility criteria and provide written consent to further contact will be sent a letter of invitation and a study participant information sheet outlining the SMOotH Study and the details of reimbursement for their travel to the clinic. Non-responders will be sent a reminder invitation two weeks later. Those willing to take part in the study will be booked into the next convenient appointment for the assessment clinic and a letter of confirmation and baseline SMOotH questionnaire mailed. The assessment clinic is expected to last approximately one hour. Participants' baseline questionnaire will be checked for completion by the research nurse at the clinic assessment.

Participants who do not attend clinic for their specified appointment will be sent another letter asking them to re-contact the research centre and to book another appointment if they still wish to participate.

On arrival at the clinic the study will be discussed with participants and written informed consent taken prior to assessment and randomisation.

Prior to assessment, all participants will undertake screening to identify possible red flags indicative of potentially serious pathology, e.g. recent trauma to the hands likely to have resulted in significant tissue damage, and acutely swollen and painful hand joints. Further screening will be carried out to determine whether the participants meet the eligibility criteria (see Table 3). This will include examination of the hand joints for features of hand OA using the ACR Classification and whether the participant has thumb base OA. Participants' availability to attend OT sessions in the next 3 months will be ascertained.

Participants who consent to participate in the study and meet the eligibility criteria will be invited to undertake a research interview and hand function assessment with a research nurse [32,33]. Assessment equipment (Jamar Dynamometer and B&L Pinch Gauge [33]) will be calibrated prior to the start of the study.

Irrespective of whether they are randomised, all participants attending the clinic will receive out of pocket expenses, an information leaflet and advice. Those who do not consent to be part of the trial or are ineligible will be asked for their consent to use the information already provided for the study and given advice to consult their GP if their hand problems continue to be troublesome. Consent forms and assessment documentation will be placed in secure storage at the research centre.

The GP will be notified whether the participant has been recruited to the trial. Any significant abnormalities identified in the clinic will be communicated to their GP via a post-clinic fax and letter.

#### Participant timeline

**Figure 1:**
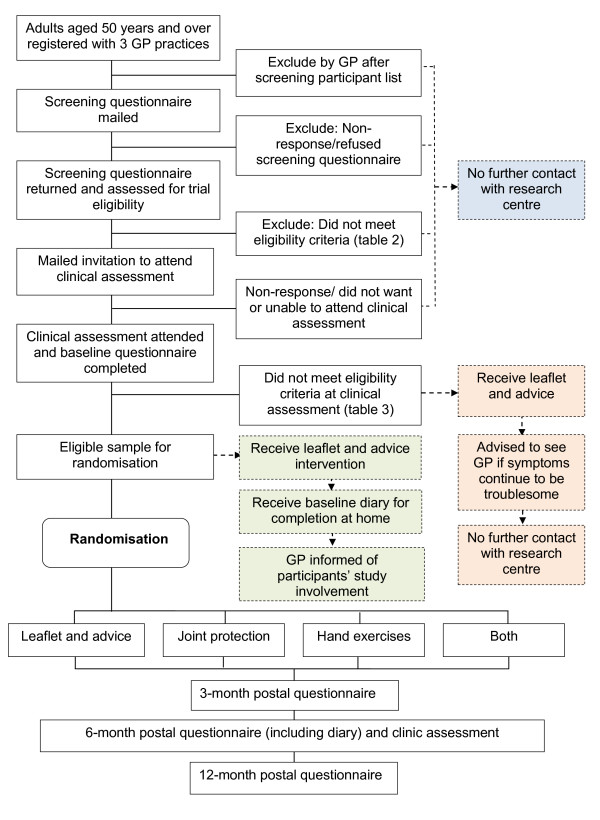
**Recruitment flow chart**.

Participant flow can be seen in Figure 1. Follow-up will be at 3 months, 6 months and 12 months after randomisation to evaluate short, medium and longer-term outcome. Six-months after attending the baseline assessment clinic, randomised participants will receive a self-administered questionnaire and an invitation to attend a brief clinical assessment of hand functional performance by a research nurse, using the same procedures as at baseline. The 3- and 12-month follow-up will be undertaken by self-administered postal questionnaire alone.

### Trial Procedures

#### Recruitment and retention strategies

Standard research centre procedures will be followed to maximise follow up. Non-responders to the health survey will receive a postcard reminder at 2 weeks followed by a second questionnaire 2 weeks later. If there is still no response, no further contact will be made. At 3- and 12-month follow-up, randomised participants will receive postcard reminders and follow-up questionnaires. Non-responders will be approached for Minimum Data Collection (MDC) 2-weeks after the second questionnaire is mailed. MDC is a shorter version of the health survey questionnaire and will be used to collect the primary outcome measure (OARSI/OMERACT responder criteria) along with date of birth, age and gender to ensure the data are provided by the intended participant. MDC is completed on the telephone, or by post where consent to telephone contact has not been given. The reminder process for baseline and 6 month nurse clinic attendance will include an initial invitation to attend the clinic, and a reminder sent two weeks later. At 6-months, if there is still no response, the follow-up questionnaire will be mailed to participants but without an invitation to attend the clinical assessment. If there is still no response after a further 2 weeks, MDC will be completed if possible. Participants failing to attend an assessment appointment will be offered a second appointment. At all stages of the trial, any reason for non-participation will be recorded, if given.

#### Consent

Only participants giving consent to further contact on the health survey questionnaire will be mailed an invitation to attend the baseline nurse clinical assessment. Face-to-face consent will also be obtained by the research nurse at the baseline clinical assessment. This consent procedure includes consent to receive follow-up mailings, randomisation to one of four treatment approaches and to attend a follow-up assessment clinic at 6 months. The research nurse will also ask participants if they give consent for the research team to access their medical records. If they do, their medical records will be tagged using an electronic computer system, to support a later review of consultation records. Participants will be informed of the right to withdraw from the study at any time and for any reason without prejudice to future care. Participants will not receive any further mailings if they wish to withdraw from the study at any time.

#### Confidentiality

Participants will be assured of confidentiality and participant details will not be made available to anyone outside the study team. GPs will be informed of their patients' willingness to be part of the study and their agreement for their inclusion will be ascertained. All participants excluded from entry into the trial at any stage will be offered standard information, on request, by the Principal Investigator and advised to consult their GP should their symptoms remain troublesome.

#### Randomisation sequence generation, allocation concealment, implementation

Randomisation will be completed at the Arthritis Research UK Primary Care Centre by administrative staff with no clinical involvement in the trial. Details of participants eligible for randomisation will be passed to Centre administrative staff by the research nurse after each research clinic. Administrative staff will enter details of those eligible for randomisation into a Microsoft ACCESS database (housed in a separate geographical location to where the clinical assessments will be conducted). Randomisation will be implemented using random permuted blocks of size 4. The blocks will be selected at random using a random number generator within the ACCESS database and will be selected separately for each GP practice. The randomised treatment of the next patient in the trial will be concealed to both administrative and OT staff until the point of randomisation. Randomisation will be completed using an allocation ratio of 1:1:1:1.

#### Blinding/masking

During the data collection phase, both the trial nurse and treating OTs will be blind to the block size used in the randomisation procedure. The research nurse will remain blind to treatment allocation until all data collection (including baseline and follow-up) has been completed. Success of blinding will be recorded by the research nurse in the clinic assessment at 6 months and during MDC over the telephone. The trial statistician will be blind to treatment allocation until the main treatment analysis has been completed.

To ensure the nurse and trial statistician remain blind to treatment allocation the following will be observed:

• The password for the database and where it is to be stored will not be known by the statistician.

• Treatment arms in the treatment database will always be stored as ABCD and the key to un-blind the treatments will only be known by the database designer and administrative staff executing the randomisation.

• The research nurse will emphasise to participants at the 6-month clinic assessment that they should not reveal what treatment they have received.

• The nurse will not have access to any of the databases. Any information passed to the research nurse (such as participant name, address and appointment time) will be done via the administrative assistant.

• Consent to participate in the trial will be done by the research nurse who will be unaware of which treatment the patient has been randomised to receive.

#### Research nurse training

To ensure standardisation, three qualified research nurses will receive training in the use of pre-defined protocols for all components of the research assessment. Training on using the protocols will be carried out prior to the clinic commencement and the protocols will be described in a Research Nurse Assessment Manual which will be available for use throughout the study period. A pilot study of the procedures using the training manual will also be undertaken.

At regular periods throughout the study, audits will be conducted to ensure quality and consistency of the research nurse assessment.

#### Study Setting

The study will be conducted in primary care. The setting will be general practices and OT outpatient facilities in Central Cheshire and North Staffordshire, UK. The GP practices, from which the participants will be recruited, cover a heterogeneous population, both socio-economically and geographically. The nurse assessment clinics will be conducted in GP practices and OT departments in local NHS hospitals. Those conducted in OT departments will be carried out in different areas and at different times to the OT interventions. Each clinic will be staffed by a research nurse who will be assisted by receptionists employed by the GP practice or NHS. Two sites in North Staffordshire and Central Cheshire will deliver all 3 OT interventions.

#### Training of Occupational Therapists

OTs delivering the intervention will initially participate in two consensus workshops to determine the most relevant, evidence-based, joint protection principles and hand exercises for use in hand OA. A literature review and analysis of evidence for joint protection and hand exercises for hand OA and RA will identify a range of principles and exercises that may be used in practice. The OTs will then be asked to identify up to 10 key joint protection and energy conservation principles considered relevant for hand OA (for example, 'distribute load over several joints', 'modify environment to support ergonomic/joint protection principles') and to identify key range of movement and strengthening exercises for the fingers, thumb and hand.

A pool of 12 OTs (2 groups of 6), with a particular interest or expertise in hand OA, will be given two days training at a local OT hospital site by the leader of the OT programme (AH). The OTs will be trained in the principles of patient education and factors affecting adherence and behaviour, including the principles of self-efficacy [34], prior to being introduced to the joint protection and hand exercise programme. The joint protection and hand exercise programmes will use Self-Efficacy Theory [34], the Health Belief Model [35], self-management cognitive-behavioural theory [36], motor learning and adult education as their basis, and will focus on addressing specific factors to support the use of joint protection techniques and hand exercises.

OTs will have the opportunity to practice teaching techniques, joint protection methods and hand exercises. Further details of the programme will be available from the Principal Investigator (KD).

#### Interventions

There will be four treatment arms to the study; leaflet and advice; joint protection delivered by an occupational therapist in a group setting; hand exercises delivered by an occupational therapist in a group setting; and joint protection combined with hand exercises delivered by an occupational therapist in a group setting.

Previous studies suggest that people with hand OA do not consult their GP very often about their symptoms and adopt their own approaches to self-management, which may or may not have beneficial effects. In order to standardise information given to participants, all eligible participants will receive information on GP headed notepaper from a research nurse prior to randomisation. Participants will be instructed to continue with their own self-management approaches, which they will be asked to record, will receive standardised advice on the use of analgesia and will be given the Arthritis Research UK leaflets 'Osteoarthritis' and 'Looking after your joints when you have arthritis' http://www.arthritisresearchuk.org. Relevant sections in the booklets will be highlighted and discussed. A leaflet on GP headed notepaper, which includes general information on looking after the joints of the hand, how to use the leaflets, and advice to consult their GP if symptoms continue to be troublesome, will be provided. Participants will also receive NICE good practice guidance [37] and advice on effective pain management with the use of paracetamol as first line analgesia, and advice on when to consult their GP. Co-interventions will be recorded and avoided during the first six months of the study.

##### Leaflet and advice

The intervention will be delivered as described above without any additional OT classes.

##### OT Interventions

Participants randomised to any one of the OT interventions will receive in addition to the above, four group sessions held once a week with 4-6 participants. A pool of 12 OTs will be trained to deliver the interventions. In order to develop rapport between participants and therapist it is planned that the same OT will conduct all four sessions. Non-trial co-interventions e.g. splinting, will be avoided during the first six months of the study and recorded if given. To reduce any potential bias, each OT will rotate throughout the interventions every three months. Rotation will be determined by the availability of the OTs to deliver the specific intervention, that is, the single component interventions, or the combined programme.

All three OT interventions will include a general introduction, education on hand OA and its management, and management of pain during everyday activities. The OT interventions will be supported by leader and participant manuals which will be used to promote treatment adherence and to standardise delivery of the OT interventions. Flipcharts will be used as teaching aids, which will identify key points to be addressed within each session, and copies of the pre-written charts will be included in the leader manuals.

Participants will be encouraged to practice techniques taught in the sessions and illustrated in the participant manual, by setting weekly action plans, homework programmes and weekly review of progress. Participants will be encouraged to continue with their own self-management approaches, which they will be asked to record in their participant manual.

###### *Joint protection classes*

The OT intervention will be based on that previously used in inflammatory arthritis and adapted for hand OA, with particular attention to hand and thumb problems [11,12]. Supervised kitchen activities will be undertaken with participants in pairs to allow demonstration and practice of new skills. Classes will be delivered over 4 group sessions (maximum 1 hour each session).

###### *Hand exercise classes*

Hand exercises to strengthen muscles and mobilise joints will be developed from those identified in the consensus workshops. These will form the basis of the exercise classes which will be demonstrated and practised with participants seated around a large table. Classes will be delivered over 4 group sessions (maximum 1 hour each session).

###### *Joint protection with hand exercises*

Participants will receive both joint protection and hand exercises over 4 group sessions (maximum 11/2 hours each session).

### Attendance protocol

The OT will be faxed a copy of the participant consent and a standard proforma prior to each session. The proforma will contain the participant identifier, the type of intervention to be delivered and the session number. At each class, the OT will confirm these details, indicate whether participants have attended and then fax the form to the study co-ordinator (SH) who will then audit adherence to the attendance protocol. The OTs will record the type of intervention received by each participant and the length of time of each treatment session on the proforma. Participants will be required to attend a minimum number of sessions. Participants failing to attend session 1 will be invited to attend session 1 of a subsequent round. Session 4 will be designed to summarise the content of the previous sessions. Participants failing to attend session 4, and not having completed sessions 2 and 3, will be invited to session 4 in a later round. Participants failing to attend session 2, 3 or both, will only be invited to repeat the missed sessions if requested by the OT or participant.

### Audit of OT interventions

In addition to the standard proforma, an audit for the group intervention will be devised based on the leader manuals. The study co-ordinator will use these to carry out random audits to assess adherence to the intervention protocol.

### Monitoring and reporting of harms

If a patient experiences an adverse event the OT concerned will inform the study coordinator by fax or telephone. The co-ordinator will investigate and record all details of the incident on an "adverse event" form. The Principal Investigator will be notified of the event, and will determine any follow-up action as required, e.g. referral to the participant's GP. All adverse events will be reported to the Data Monitoring Committee and the Trial Steering Committee.

### Equipment

All OT sites will be provided with a standardised equipment package for the delivery of the joint protection and exercise programmes. An equipment inventory is available on request from the study co-ordinator.

### Electronic OT mailing list

In order to enhance protocol adherence and to offer support to the OTs involved in the trial, the Principal Investigator and study co-ordinator will set up a shared electronic mailing list for participating therapists.

### Pilot study

Up to 6 participants will be invited to attend a pilot study of the OT intervention. These participants will be members of the Centre Research Users Forum and will have a history of hand OA. The pilot study will be based on the combined programme of hand exercises and joint protection, and will take place at a local OT Department. The study will test processes and procedures, and any further amendments to the content of the intervention will be made prior to the commencement of the main trial.

### Data collection management and analysis

#### Primary outcome measure

Study outcomes are documented in Tables 4 and 5, and are based on previously validated measures [38,39]. The primary outcome will combine pain and function subscales of the AUSCAN [31,40] and global assessment of improvement [41] to determine a 'responder' using the OARSI-OMERACT criteria [42] at 6 months post randomisation. Response options for the AUSCAN items are on a 5-point scale ranging from none to extreme, and for the purpose of this study the AUSCAN validated for use in older adults with hand pain in the population will be used [40]. Global assessment of improvement is on a 6-point scale ranging from completely better to much worse.

**Table 4:** Secondary outcome measures

Outcome	Measurement Scale	Time points
AUSCAN pain [31,40]	0-20	HS, 0Q, 3, 6Q, 12, +
AUSCAN stiffness [31,40]	0-4	HS, 0Q, 3, 6Q, 12, +
AUSCAN function [31,40]	0-36	HS, 0Q, 3, 6Q, 12, +
Total AUSCAN [31,40]	0-12	HS, 0Q, 3, 6Q, 12, +
Pain severity in the last 3 days	NRS: 0-10	0NA, 3, 6NA, 12
Severity of participant nominated worse problem in the last 3 days [44]	NRS: 0-10	0NA, 3, 6NA, 12
Satisfaction with hand function in the last 3 days	NRS: 0-10	0NA, 3, 6NA, 12
Power grip (JAMAR dynomometer) [33]	lbs	0NA, 6NA
Pinch grip (B&L pinch gauge) [33]	lbs	0NA, 6NA
Grip-ability test (GAT) [32]	Timed (seconds)	0NA, 6NA
Short-form 12 (SF-12): physical and mental health component scores [49]	0-100	HS, 0Q, 3, 6Q, 12
Arthritis self efficacy for pain [50]	1-10	HS, 3, 6Q, 12
Global assessment of change in hand problem [41]	Completely recovered/much better/better/no change/worse/much worse	3, 6Q, 12, +

NRS = Numerical rating scale; HS = Baseline Health Survey; 0Q = Baseline Questionnaire; 0NA = Baseline nurse assessment; 3 = 3-month questionnaire; 6Q = 6-month questionnaire; 6NA = 6-month nurse assessment; 12 = 12-month questionnaire; + = included in minimum data collection.

**Table 5:** Tertiary outcome measures

Outcome	Measurement Scale	Time points
*Demographic variables*		

Age	Years	HS, 0Q, 3, 6Q, 12, +
Gender	Female/Male	HS, 0Q, 3, 6Q, 12, +
Marital status	Married/Separated/divorced/widowed/cohabiting/single	HS
Employment status	Working full time/working part time/working full time in the home/unemployed or seeking work/not working due to ill health or disability/student/retired	HS
Social class [65]	Higher managerial/Higher professional/Lower managerial or professional/Intermediate occupations/Self employed/Lower supervisory or technical/Semi-routine/Routine	HS, 0NA
Age when left school	Years	HS
Leave school to go to full-time education or university	Yes/No	HS
Age when finished full time education	Years	HS
Gained qualifications through study as an adult	Yes/No	HS
Ethnic origin	White UK or European/AfroCaribbean/Chinese/Asian /African/Other	HS
Height	Feet and inches or centimetres	HS
Weight	Stones and lbs or kilograms	HS

*Measures to define trial inclusion/exclusion *		

Hand pain in the last year	Yes/No	HS
Pain, aching or stiffness in your hands in the last month [30]	No days/Few days/Some days/Most days/All days	HS, 0Q, 0NA, 3, 6NA, 12
Seen OT or PT for hand problem in last 6-months	No/Right hand only/Left hand only/Both hands	HS, 0NA^1^
Injured hands badly enough to see a doctor in last 6 months	No/Right hand only/Left hand only/Both hands	HS, 0NA^1^, 6NA
Had a hand operation in the last 6 months	No/Right hand only/Left hand only/Both hands	HS, 0NA^1^, 6NA
Joint injection (fingers, thumbs or wrist) in the last 6 months	No/Right hand only/Left hand only/Both hands	HS, 0NA^1^
Clinical red flags (e.g. swollen painful hot hands or recent trauma to the hands)	Yes/No	0NA, 6NA
Clinical assessment for hand swelling, nodes, enlargement or deformity	Yes/No for joints required to apply the ACR criteria for hand OA (ref)	0NA

*Characteristics of hand problem*		

Handedness	Right/Left/Both	HS, 0NA, 6NA
Hand problems in the last year	Yes/No	HS
Hand pain location in the last year	Right/Left/Both	HS, 0NA^2^, 6NA^2^
Hand pain manikin [43]	Hand area shaded to represent pain	0Q, 3, 6Q, 12
Hand that gives most problem	Right/Left/No difference	HS, 0NA, 6NA
Number of days with hand pain in the last year [66]	Less than 7 days/1 to 4 weeks/> 1 month and < 3 months/3-months or more	HS
Hand pain severity in the last month	NRS: 0-10 (no pain to pain as bad as could be)	0Q
Bothersomeness of hand problems (adapted from [67])	Not at all/slightly/moderately/very much/Extremely	0Q, 3, 6Q, 12
Thumb pain during activity in the last month	Yes/No	0NA, 3, 6NA, 12
Ability to make a fist [68]	Right: yes/no/unable; Left: yes/no/unable	0NA, 6NA
Length of time with a hand problem	HS-years 0NA-Separate response for right and left hand < 12 months/1 to 5 yrs/5 to 10 yrs/10+ yrs	HS, 0NA
Previous hand surgery	Operation details, which hand, timing (< 1 yr ago/1 to 5yrs ago/5 to 10yrs ago/10+ yrs ago)	0NA, 6NA^3^
Previous hand injury	Injury details, which hand, timing (< 1 yr ago/1 to 5yrs ago/5 to 10yrs ago/10+ yrs ago)	0NA, 6NA^3^
Past or present job involved excessive use of hands	Yes/No	HS
Past or present hobbies or pastimes involved excessive use of hands	Yes/No	HS
Global assessment of change in hand pain since baseline [41]	Completely recovered/much better/better/no change/worse/much worse	3, 6Q, 12
Global assessment of change in ability to use hands since baseline [41]	Completely recovered/much better/better/no change/worse/much worse	3, 6Q, 12

*Body pain and shoulder function*		

Pain elsewhere (body manikin)	Body area shaded to represent pain lasting for a day or longer in the last 4 weeks	HS, 3, 6Q, 12
Ability to put hands behind head [68]	Right: yes/no/unable; Left: yes/no/unable	0NA, 6NA

*Perception, impact and quality of life*		

Illness perceptions	Subset of questions from the illness perceptions questionnaire revised (IPQ-R) [45,51]	HS, 0Q, 3, 6Q, 12
Participation restriction (selected questions from [46])	All the time/most of the time/some of the time/A little of the	0Q, 3, 6Q, 12
- Self-care needs met as and when wanted	time/None of the time	
- Home looked after as and when wanted		
- Belongings looked after as and when wanted		
Hand problems make you feel frustrated in the last month [45]	All days/most days/some days/few days/no days	0Q, 3, 6Q, 12
Adaptation behaviours		0NA, 6NA
- Use gadgets	Yes/No to each question	
- Help from another person		
- Avoidance		
- Find a different way of doing something		
- Stopping/reducing activities		
- Things take longer		
- Other (please state)		
Quality of life: EuroQol-EQ5-D [47,48]	(-0.59-1)	0Q, 3, 6Q, 12

Intervention evaluation		

Satisfaction with care received for hand problem	Very satisfied/Quite satisfied/no opinion/not very satisfied/not at all satisfied	3, 6Q, 12
Treatment sessions: Number of appointments	Too many/About right/Not enough/I did not attend any appointments	3
Treatment sessions: Length of each visit	Too long/About right/Too short/I did not attend any appointments	3
Hand exercise frequency [69]	Never/Once a week/Twice a week/Three times a week/Four times a week/Five times a week/Six times a week/Once every day/Twice every day	6Q, 12
Hand exercise frequency in last week [52]	Never/Almost never/Sometimes/Fairly often/Very often/Always	0Q, 3, 6Q, 12
Hand exercise duration [69]	< Five minutes/5-10 minutes/10-15 minutes/15-30 minutes/30 minutes+/I don't do hand exercises	6Q, 12
Behaviour change in last week: energy conservation/fatigue [52]	(1-6) i.e. average of 5 items each rated as: Never/Almost never/Sometimes/Fairly often/Very often/Always	0Q, 3, 6Q, 12
- Regular breaks		
- Breaking up tasks		
- Pacing of activities - Swapping between light and heavy tasks		
- Maintaining good posture whilst sitting, standing, lifting objects or moving about		
Behaviour change in last week: joint protection use [52]	(1-6) i.e. average of 5 items each rated as: Never/Almost	0Q, 3, 6Q, 12
- Use two hands to carry things	never/Sometimes/Fairly often/Very often/Always	
- Avoid gripping or pinching things tightly		
- Change the way everyday activities are completed		
- Use gadgets/labour-saving devices		
- Use stronger, larger joints		
Behaviour change in last week: carry on working through the pain when doing everyday activities [52]	Never/Almost never/Sometimes/Fairly often/Very often/Always	0Q, 3, 6Q, 12

*Health care use and co-interventions*		

Self-report	Self-help remedies, contact with NHS and private healthcare, over the counter medicines, use of hand splints	HS, 0NA, 6Q, 6NA, 12
GP consultation download	Number of follow-up visits to the GP, prescription of medication including NSAIDs and referral for other treatment such as surgery	Continually collected data

*Nurse audit questions *		

Did the participant un-blind you during the assessment?	Yes/No	6NA
If yes, what did the participant say and could it have been avoided?	Text	6NA
If yes, what treatment arm do you think the patient is randomised to	Leaflet and advice/Had OT, but not sure which OT intervention/Had OT, joint protection/Had OT, hand exercises/Had OT, joint protection and hand exercises	6NA

NRS = Numerical rating scale; OT = occupational therapist; PT = Physiotherapist; HS = Baseline Health Survey; 0Q = Baseline Questionnaire; 0NA = Baseline nurse assessment; 3 = 3-month questionnaire; 6Q = 6-month questionnaire; 6NA = 6-month nurse assessment; 12 = 12-month questionnaire; + = included in minimum data collection; 1 = time frame last month; 2 = refers to current hand problem; 3 = time frame last 6 months.

Minimum data collection at each follow-up data collection stage will attempt to capture the primary outcomes, AUSCAN and global change scores, in the event of non-response to the mailed follow-up questionnaire.

#### Secondary and tertiary outcome measures

##### Self-reported questionnaire at baseline, 3, 6 and 12 months

Individual subscales of the AUSCAN (pain, stiffness and function), hand pain manikin [43], average pain severity over the past 3 days (0-10 numerical rating scale), severity rating of participant nominated main functional problem over the past 3 days (0-10 numerical rating scale) [44], satisfaction with hand function over the past 3 days (0-10 numerical rating scale), side effects of treatment and adverse events, co-interventions (from the medical record download: follow-up visits to the GP, prescription of medication including NSAIDs and referral for other treatment such as surgery and from self-reported questionnaires: self-help remedies, contacts with private healthcare, over the counter medicines, use of hand splints), frustration related to hand disability [45], pain elsewhere (pain manikin), participation restriction [46], health-related quality of life using the EuroQol EQ-5D [47,48] and SF12v2 [49], satisfaction with care (3 and 6 months), Arthritis Self Efficacy pain subscale [50], Illness Perceptions Questionnaire-Revised (IPQR) modified for hand OA [45,51] and self-reported behaviour change using selected questions [52].

##### Clinical assessment at baseline and 6 months only

grip strength (JAMAR) [33], pinch strength (B & L pinch gauge) [33], functional performance using the grip ability test (GAT) [32]. (See Tables 4 and 5).

#### Diary

All participants randomised to the trial will be given a diary to complete at baseline (nurse clinical assessment) and at 6 months (the primary end point). The diary is based upon the Activity Record (ACTRE) for patients with musculoskeletal disorders [53,54]. The diary aims to capture hand pain and functional limitation experienced when carrying out main activities for each half hour during a typical weekday and a weekend day, along with any rest periods taken during the activities. For each main activity, in each half hour period, participants will rate their hand pain and hand disability on a 0-3 scale, where 0 represents 'no hand pain/disability' and 3 represents 'a lot of hand pain/disability'. The 6-month diary will also include open ended questions to ask participants if they feel they have benefitted from taking part in the study and if not what they feel would have been beneficial. Participants will also be invited to make any additional comments if they wish.

#### Target sample size

The main study sample size calculation will be based on the comparison of participants receiving and those not receiving hand exercises. The calculation would be identical for the comparison of joint protection versus no joint protection, as hand exercises and joint protection are assumed equally effective and independent treatments [55].

In participants not receiving hand exercises 50% will receive a leaflet and advice, and 50% will receive joint protection. We estimate that 25% of participants in the leaflet and advice group will improve using the OARSI-OMERACT responder criteria and 45% will improve in the joint protection group [42,56]. This gives a combined improvement of 35% in participants not receiving hand exercises, assuming equal allocation of participants between treatment groups.

Published information is not available to define a minimum clinical important difference for the primary outcome measure. Therefore, after a consensus discussion with the OTs we estimate this at 20%, and hence the estimate of improvement in the group who receive hand exercises to be 55% (i.e. 35% + 20%). To detect a difference of 20% or larger between participants receiving and those not receiving hand exercises, with 80% power and alpha of 5%, a total of 212 participants with data at baseline and at 6 months are required. To allow for a 15% drop-out over the 6 months post randomisation period, 252 participants will be randomized, i.e. 63 per treatment arm.

#### Statistical methods, between group comparisons, handling of non-adherence and missing data

The main statistical analysis will be based on reporting guidelines for the design and conduct of factorial trials [55] and will be conducted for all primary and secondary outcomes. The main treatment analysis will be conducted blinded to treatment allocation and will be analysed on an intention to treat basis with all randomised participants retaining their original randomised group. Outcome measures that are continuous will be analysed using analysis of covariance (ANCOVA); for binary outcomes, logistic regression will be used. The data will be analysed at 3, 6 and 12 month follow-up, however, 6 months is the primary end point for the study.

An initial treatment model will be fitted (for each primary and secondary outcome and end-point) to predict the outcome of interest and will include the two treatment effects of interest: no joint protection versus joint protection; no hand exercises versus hand exercises, and their interaction. If the interaction term is not statistically significant (p > = 0.05) it will be dropped from the model. The model will be re-run, and the treatment effects for joint protection and hand exercises determined individually from this model, either as mean differences or odds ratios with associated 95% confidence intervals, as appropriate. If the interaction term is statistically significant (p < 0.05), the effect of joint protection and hand exercises will be evaluated from a model with treatment represented as a 4-level variable (i.e. leaflet and advice, joint protection, hand exercises, joint protection and hand exercises) and the reduced statistical power of this model noted. This model will also be used as a secondary analysis to compare the effectiveness of the individual treatments to the leaflet and advice arm.

All analysis models will be adjusted for the baseline value of the outcome of interest (with the exception of the OARSI/OMERACT responder criteria which is not computable at baseline) and also for age, gender, social class, length of time with a hand condition and general practice (covariates defined a priori as those that may influence treatment outcome). Missing data will be imputed using the multiple imputation routines in STATA version 11.0 [57].

A sensitivity analysis will be completed to examine the effectiveness of joint protection and hand exercises for those participants attending all four treatment sessions. This analysis will only be completed if there are sufficient participants attending all four treatment sessions. Treatment concordance will also be evaluated descriptively by (self-reported) frequency and duration of hand exercise completion at 3-, 6- and 12-month follow-up.

Generalisability of the trial findings and the success of the randomisation procedure will be explored descriptively by comparing key characteristics of participants at recruitment and each follow-up stage and for each randomised treatment arm. No interim analyses will be planned during the trial follow-up period.

#### Health economics

The purpose of economic evaluation is to inform decision makers about competing claims for health care resources. Uncontaminated estimates of costs and effects of alternative treatments are the key parameters for the provision of cost effectiveness evidence and, accordingly, the clinical analytic framework for factorial design randomised controlled trials is not suitable because of the combination of treatment regimens.

The estimation of cost-effectiveness within this 4-arm study will focus on the principles of dominance and extended dominance. Dominance is a straightforward concept; if an intervention is less effective and more costly than at least one of its comparators, it is not for further consideration with regard to the estimation of cost-effectiveness. Extended dominance is applied in incremental cost-effectiveness analysis when an intervention is less effective and more costly than a linear combination of two other strategies; the purpose is to remove from consideration those strategies whose costs and benefits are improved by a mixed strategy of two other alternatives [58]. The practical application of cost-effectiveness analysis is to compare an intervention with the next most effective strategy; failure to remove all dominated or extendedly dominated strategies may lead to comparisons that are not with the next best alternative but with irrelevant alternatives.

In the base case analysis, the estimation of costs relating to the UK National Health Service (NHS) will be based on responses to health care resource use questions within the 6-month and 12-month postal questionnaires; responses will be aggregated to generate a 12-month cost estimate for each responder. The resource use questions will capture details covering a broad range of health care resources, including prescribed medications, primary care and secondary care (inpatient and outpatient) attendances, treatments and investigations. The primary unit of benefit is the quality-adjusted life year (QALY), calculated by applying area-under-the-curve techniques to EuroQol EQ-5D index scores at baseline, 3 months, 6 months and 12 months [59]. The EQ-5D is a generic health status measure that provides utility values for all possible responses to the 5-dimension questionnaire based on health state valuations elicited from a large representative sample of the UK population [60]. The values range from 1.00 (no problems on all dimensions) to -0.59 (severe or extreme impairment on each dimension). Accordingly, the maximum number of QALYs per patient is equal to 1 (equivalent to 12 months spent in full health), with QALYs less than 1 reflecting less than perfect health. Following the identification of appropriate pair-wise comparisons through extended dominance principles, differences in costs and QALYs will be expressed using the incremental cost-per-QALY ratio. This ratio measure provides an estimate of the additional cost necessary to generate one additional QALY. Multiple imputation techniques will be used to deal with missing EQ-5D scores and resource use data, ensuring that all eligible trial participants are included in the base case economic evaluation [61,62].

Probabilistic sensitivity analysis will address uncertainty around the incremental ratio through the application of bootstrap techniques to generate cost-effectiveness planes and acceptability curves [63,64]. Further sensitivity analysis will explore the robustness of the results to variation in key parameters and methodological techniques; namely, the adoption of alternative costing methodologies (e.g. 'generic' verses 'hand OA-specific' health care resource use), a broader analytic perspective that incorporates costs beyond those attributable to the UK NHS, a complete-case analysis to consider the implications of missing data, and the impact of using different generic health status measures to provide utility values.

#### Trial monitoring

The research centre's independent Data Monitoring Committee (DMC) will monitor the study 6-monthly and reports will be written in line with Arthritis Research UK recommendations (http://www.arthritisresearchuk.org). The independent DMC has also agreed to act as the trial steering committee.

#### Research Ethics

Ethical approval was obtained from the Central Manchester Research Ethics Committee, UK on 21^st ^February, 2008 [ref number 07/H1008/235]. Any subsequent amendments will be reported in the DMC reports.

## Discussion

There is limited evidence for the clinical and cost effectiveness of OT approaches in the management of OA despite the important role that OTs play in the treatment of people with hand OA. Joint protection and hand exercises have been proposed by European guidelines for hand OA [13]. However, the clinical and cost effectiveness of each intervention and the combined approach is unknown.

This protocol outlines the SMOotH study, a multicentre two-by-two factorial randomised controlled trial in community-dwelling older adults. The aims are (i) to compare the effectiveness of joint protection delivered by an OT with no joint protection, (ii) to compare the effectiveness of hand exercise delivered by an OT with no hand exercises and (iii) to determine which of the four management approaches explored within the study (leaflet and advice, joint protection, hand exercises, or joint protection and hand exercise combined) provides the most cost-effective use of health care resources.

Findings from this study will contribute to the cost-effective evidence based management of hand OA and to existing recommendations published by EULAR.

### Role of individual parties

Principal investigator: Krysia S. Dziedzic; Study Coordinator: Susan Hill; Trial Statistician: Elaine Nicholls; Leader of the OT programme: Alison Hammond; Informatics Manager: Tracy Whitehurst; Centre Operations Manager: Jo Bailey; Health Economist: David G.T. Whitehurst, Sue Jowett; Trial Steering Committee and Data Monitoring Committee: Chris Roberts (Chair), James Selfe, Christina Jerosch-Herold and Richard McManus; Study Design: Helen Myers, Charlotte Clements, June Handy, Rhian W. Hughes, Elaine Thomas, Elaine M. Hay.

## Abbreviations

AUSCAN: Australian/Canadian Osteoarthritis Hand Index; DMC: Data Monitoring Committee; EULAR: European League Against Rheumatism; GP: General Practitioner; NICE: National Institute for Clinical Excellence; OA: Osteoarthritis; OARSI/OMERACT: Osteoarthritis Research Society International/Outcome Measures in Rheumatological Clinical Trials; OT: Occupational Therapy/Occupational Therapist; PT: Physiotherapist; RA: Rheumatoid arthritis.

## Competing interests

The authors declare that they have no competing interests.

## Authors' contributions

All authors participated in the design of the study and drafting the manuscript. All authors read and approved the final manuscript.

## Pre-publication history

The pre-publication history for this paper can be accessed here:

http://www.biomedcentral.com/1471-2474/12/156/prepub
